# Contributions to Ear-Medicine

**Published:** 1847-07

**Authors:** 


### Art. II.

*Beiträge zur Ohrenheilkunde von* Dr. Wilhelm Kramer, Sanitäts-Rath. Nebst 19 statistischen Tabellen.—*Berlin,* 1845.

*Contributions to Ear-Medicine.* By Dr. William Kramer, &c. With 19 Statistic Tables.—*Berlin,* 1845. 8vo, pp. 314.

The principal of these contributions, viz., the statistics of the diseases of the ear, the author tells us, are the result of fifteen years' careful observation, and are entirely practical; the utmost possible accuracy of diagnosis and simplification of treatment, the only true aims of the practitioner, having been alone kept in view. In these respects, indeed, the author wishes the present contributions to be considered as a necessary complement to his work on the Nature and Treatment of the Diseases of the Ear, published in 1836, and noticed in the number of this Review for Jan. 1837.

The materials for the statistics are 2000 cases of diseases of the ear, all of which Dr. Kramer tells us he explored, by means of the speculum auris, the Eustachian tube catheter, the air-douche, &c., and carefully registered in his journal the results thus obtained as well as all that relates to age, sex, and residence of the patient; the date of origin and causes of the disease; the existence or absence of tinnitus and other symptoms; the hearing distance for each ear; the treatment, if any, adopted before the patient's application to the author; the author's own treatment; duration, and event.

In a system of the diseases of the ear, it is, says Dr. Kramer, "my opinion that those diseases only should be admitted which, in consequence of the peculiar structure of the organ affected, and the peculiarity of its function as an organ of sense, are accompanied by such peculiar symptoms, that they are thereby distinguishable from all other diseases of the body."

In the classification of the diseases of the ear, Dr. Kramer says he has followed that laid down in his treatise above referred to, the more decidedly, as it is founded on the very simple and natural principle of anatomical difference of the structures composing the organ of hearing; the morbid states of which, moreover, present such definite characters, that in the course of fifteen years not a single case has occurred to him which does not find its place in his system, in a natural and unforced manner.

In the present work, for the sake of more fully carrying out his principle of classification, he goes on to say, he has changed the nomenclature of the diseases of the auricle as follows. Instead of the names *erysipelatous inflammation, scirrhous degeneration* and *boils,* he employs respectively *inflammation—of the epidermis, of the dermis,* and of *the cellular tissue of the auricle.* For the same reason, *erysipelatous inflammation of the auditory passage* he now calls *inflammation of the epidermis of the auditory passage.*

It is, doubtless, a good principle in defining and classifying diseases to make particular reference to the structure affected, but it ought not to be pushed too far. Diseases are seldom entirely confined to a single structure, and a secondary disease is often very different in its nature and even seat from the primary which gave rise to it, and cannot, therefore, well be described under the head of the latter. Dr. Kramer, we must confess, in pushing the simplicity of his system so far as he does, loses sight of these considerations, and is thus often betrayed into the very hypothetical and speculative admissions which he takes so much pains to protest against.

In substituting the name of "inflammation of the epidermis" for "erysipelatous inflammation," Dr. Kramer certainly not only pushes his principle too far, but also makes hypothetical and speculative admissions not only uncalled for by, but positively opposed to, practical utility. Everybody knows what is meant by erysipelatous inflammation ; but the name inflammation of the epidermis, even if we were for a moment disposed to admit it as correct, could be admitted only as a generic not as a specific name. The name "scirrhous degeneration" Dr. Kramer was right to drop, but we reject the name he would substitute for it, inflammation of the dermis of the auricle. There are various inflammations of the dermis. The disease Dr. Kramer refers to appears to be crustaceous herpes, or an affection resembling elephantiasis. Again, the substitution of the name of "inflammation of the cellular tissue" for boils, thus doing away with the distinction between boils and abscesses, is by no means warranted. What has just been said of the substitution of the name of inflammation of the epidermis for erysipelatous inflammation in the case of the auricle, is, of course, applicable to the erythema of the skin of the auditory passage, in which accumulation of wax has its origin.

The way in which Dr. Kramer describes and arranges this same accumulation in the auditory passage, under the head of "inflammation of the epidermis of the auditory passage," illustrates the absurdity to which principles carried out to an extreme will lead.

In articles on the ear in the Numbers of this Review for Jan. 1837, and July 1839, we took occasion to refer to an opinion of Mr. Swan's, that in a great proportion of the cases commonly put down as nervous deafness, the auditory nerves are not affected ; but that the deafness is owing to a thickened state of the lining membrane of the cavity of the tympanum, involving the tympanic nerves, and that the effect of Dr. Kramer's treatment of nervous deafness by means of vapours of acetic ether was more explicable according to this view of the pathology, than according to that generally received and adopted by Dr. Kramer.

We, however, remarked that perhaps the thickened state of the lining membrane of the tympanum, which Mr. Swan had observed in his dissections of the ears of habitually deaf persons, and which has also been observed in numerous other cases on record; and its consequent effect on the fenestræ, ossicles, and membrana tympani, is sufficient to explain the deafness without having recourse to Mr. Swan's supposition, that the deafness in such cases is owing to implication of the tympanic nerves.

This view of ours, which was founded on numerous well reported cases and dissections on record, has been pretty generally admitted in this country, and, in additional support of it, Mr. Toynbee has made known a very large number of dissections in which he found thickening of the lining membrane of the tympanum.

We, at the same time, did not deny that many cases of habitual deafness are actually nervous; that is, cases of disease of the auditory nerve or brain; and that many are also affections of other structures in the labyrinth.

In the pages before us, Dr. Kramer labours hard to refute this view and to maintain his own, but we must say his arguments are none of the soundest.

In the first place he is altogether mistaken in supposing that the thickened state of the mucous membrane lining the tympanum, which we consider to be the morbid condition in a large number of cases of habitual deafness, is necessarily at all a stage of catarrhal inflammation of the middle ear; and, therefore, his elaborate comparison of catarrhal inflammation of the middle ear, and his nervous deafness, at pp. 190-1, as regards their characters and attendant circumstances, has no bearing at all on the question.

"The lining membrane of the tympanic cavity being fibro-mucous, at once periosteum and mucous membrane—inflammation of it will vary in its nature and consequences. It may be at one time simply the catarrhal affection of a mucous membrane; at another, the violent purulent *otitis interna*, in which the surrounding osseous textures become so readily involved. The morbid state of the lining membrane of the middle ear I am attempting to illustrate, appears to be of the nature of periosteal swellings. On dissection, the membrane which is naturally very thin and transparent, has been found thickened and swollen, sometimes to such a degree as to bury the ossicles and overlap the fenestræ."*

He also betrays rather confined views as to the pathology of nervous deafness. Any disease entitled to the name of nervous deafness, ought to be analogous to amaurosis. Now, as the morbid conditions of the paralysis of the optic nervous apparatus in amaurosis may vary in their nature and have different seats, so also may the morbid conditions of the paralysis of the auditory nervous apparatus.

TABLE I.—*General view of the diseases of the ear.*

Of the 2000 cases, 5 or 1-400th were cases of disease of the auricle; 281 or 1-7th, cases of disease of the auditory passage; 442 or 1-4th and a half, of the membrana tympani; 198 or 1-10th, of the middle ear; 1028 or half, nervous deafness, as understood by Kramer; and 46 or 1-43d, deaf-dumbness.

*Auricle.* Of the five diseases of the auricle, 1 was erysipelas; 3 induration and thickening; 1 boil or abscess. Disease of the auricle, however,

* Cyclopædia of Practical Surgery, art. Ear and Hearing, Diseases of.

Kramer admits may be more frequent than is here represented, as they are not of a nature to have come under his especial notice.

*External auditory passage.* Of the 281 diseases of the external auditory passage, 213 were cases of concretions of wax from erythematous inflammation of the skin of the passage; 51 cases of catarrhal inflammation of the passage, or of what Dr. Kramer calls inflammation of the glandular skin; 9 were phlegmonous inflammation, and 8 periostitis and caries. The last three diseases of the auditory passage, viz., catarrhal inflammation, phlegmonous inflammation, and periostitis with caries, are accompanied by discharge from the ear; but the cases of discharge from the ear depending on them, in number 68, form but a small proportion, 1 in 7 of the whole number of 510 cases of discharge from the ear. In the remaining 6-7ths the discharge from the ear depends on inflammation of the membrana tympani.

*Membrana tympani.* The 442 cases of disease of the membrana tympani, were cases of inflammation and its consequences; and of these 45 were cases of acute inflammation, and 397 cases of chronic inflammation.

The diseases of the membrana tympani are twice as numerous as all the diseases of the auditory passage taken together, and as above stated the source of about 6-7ths of all discharges from the ear.

Nearly every fourth case of dullness of hearing has for its cause inflammation of the membrana tympani or its consequences.

In inflammation of the membrana tympani, the auditory passage in general remains unaffected.

*Middle ear.* Of the 198 cases of disease of the middle ear, 164 were catarrhal inflammation of the mucous membrane with muculent obstruction; 28 inflammation with stricture of the Eustachian tube; 2 obliteration of the Eustachian tube; and 4 inflammation and abscess of the cavity of the tympanum.

Catarrhal inflammation of the middle ear with muculent obstruction thus forms 1-12th of the whole 2000 cases of ear disease, and about 4-5ths of all the diseases of the middle ear.

This frequency of catarrhal inflammation of the middle ear is less than might have been expected from the proximity of the middle ear to the nose and throat, which are frequently the seat of catarrhal affections. It is, indeed, not uncommon for a slight degree of deafness to occur in ordinary nasal catarrh, but it in general subsides along with the latter; in such cases it is probable that the orifice of the Eustachian tube merely is affected. Very rarely is the cavity of the tympanum involved, but when it is, it will remain so long after the subsidence of the nasal and guttural affections.

Stricture of the Eustachian tube in a decided degree occurs rarely, 1 : 71 of the whole 2000 cases, and 1 : 7 of all the diseases of the middle ear.

Complete closure of the Eustachian tube is very rare.

Inflammation and abscess of the cavity of the tympanum. Dr. Kramer thinks that this does not occur as an idiopathic acute disease except when traumatic; but that it always supervenes on a chronic inflammation of the lining membrane of the tympanum, existing in connexion with chronic inflammation and perforation of the membrana tympani.

*Internal ear.* Of the 1074 cases of disease of the internal ear, 1028 or rather more than half of the whole 2000, are put down as cases of nervous deafness; the remaining 46 were cases of deaf-dumbness.

Dr. Kramer concludes his commentary on Table i with the following observations on the exploration of the ear in order to a diagnosis:

"If we now take a glance at the diagnostical relations of the diseases of the ear which have been thus passed in review, it will be found that those which can be at once recognized on simple examination, viz. the diseases of the auricle, are the very fewest in number and the least important. All the other diseases of the ear require for their diagnosis special means of exploration. For the diseases of the external auditory passage and of the membrana tympani, the employment of the speculum auris in clear sunshine is necessary; but by means of them the diagnosis of the diseases in question may be made with as much certainty as the diagnosis of any superficial affection of the skin. Without these means of exploration, however, it is quite impossible to acquire, even in the most remote degree, a correct idea of the seat and peculiar nature of the diseases of the external auditory passage and of the membrana tympani, and just as little to strike out the right way to their successful treatment. According to the above table, the diseases of the external auditory passage and of the membrana tympani constitute about a third (728) of all diseases of the ear, and that of this number the diseases of the membrana tympani constitute two thirds (442). The importance of the membrana tympani to the integrity and function of the organ of hearing is especially evident from Tables v and xiii, even in the absence of more decisive proof.

"The remaining two thirds of all diseases of the ear (1272) have their seat in parts of the organ which lie behind the membrana tympani, and are thus completely withdrawn from ocular observation. The speculum auris here assists merely in the determination that no disease of the auditory passage or membrana tympani exists, and that therefore the seat of the disease must be looked for in the middle or internal ear. In this case the employment of the Eustachian tube catheter is the only means, in order, by blowing through it, or by sending in a stream of air from the air-press, or by the introduction of a catgut string, a whalebone or ivory probe, to get acquainted with the state of the Eustachian tube and cavity of the tympanum, and from thence to conclude as to the state of the auditory nerve. Much, indeed, here depends on an experienced, fine touch and hearing, especially on the latter, in judging of the sound produced by the air streaming into the tympanum from the Eustachian tube catheter. The hearing, it is to be admitted, does not afford us such trustworthy indications of certain morbid states as vision; but this is by no means any reason for rejecting the assistance which those means afford us in investigating deeply-seated diseases of the ear.

"From all this it will be clear to every unprejudiced person, that no disease of the ear, which has not its seat in the auricle, should be treated, until it has been most carefully explored by means of the speculum, and in the cases requiring it, by means of catheterism, &c., also, and its nature fully ascertained." (p. 26.)

Table ii.—*Of the relative frequency of diseases of the ear in the two sexes.*

Of the subjects of the whole 2000 cases 1274 were males and 726 females = $1\frac{5}{7}$ males to 1 female.

*Auricle.* Of the five diseases of the auricle, the subjects of the one case of erysipelas and the one case of boil were males, and the subjects of the three cases of eczematous disease were females.

*External auditory passage.* Of the subjects of the 281 diseases of the external auditory passage, 207 were males and 74 were females. Of the subjects of the 213 cases of accumulation of wax from erythematous inflammation of the skin of the auditory passage, 161 were males and 52

females = 3 : 1. Of the subjects of the 51 cases of inflammation of the tegumentary lining of the auditory passage and ceruminous glands, or as Dr. Kramer calls it, inflammation of the glandular skin, 34 were males and 17 females = 2 : 1. Of the subjects of the 9 cases of phlegmon 6 were males and 3 females = 2 : 1. Lastly, of the subjects of the 8 cases of periostitis and caries of the auditory passage 6 were males and 2 females = 3 : 1.

*Membrana tympani.* Of the subjects of the 442 cases of disease of the membrana tympani, 311 were males and 131 females = $2\frac{1}{3}$ : 1. Of the subjects of the 45 cases of acute inflammation 33 were males, and 12 females = 3 : 1. And of the subjects of the 397 cases of chronic inflammation 278 were males, and 119 females = $2\frac{1}{3}$ : 1.

*Middle ear.* Of the subjects of the 198 cases of the diseases of the middle ear admitted by Dr. Kramer, 141 were males, and 57 females. Of the subjects of the 164 cases of muculent obstruction of the middle ear, 117 were males, and 47 females = $2\frac{1}{2}$ : 1. Of the subjects of the 28 cases of stricture of the Eustachian tube, 20 were males, and 8 females = $2\frac{1}{2}$ : 1. Of the subjects of the 2 cases of obliteration of the Eustachian tube, one was a male, and the other a female. Of the subjects of the 4 cases of otitis interna 3 were males, and 1 a female.

*Internal ear.* Of the subjects of the 1028 cases of Dr. Kramer's nervous deafness, 581 were males, and 447 females = $1\frac{1}{3}$ : 1. And of the subjects of the 44 cases of deaf-dumbness, 32 were males, and 14 females = 2 : 1.

Table III.—*Of unilateral and bilateral affections of the ears.*

In determining whether one ear only or both are at the same time affected, the patient's account alone must not be relied on, for very often the patient is under the impression that he hears quite well with one ear when he only hears *not so ill* as with the other;—or he complains of discharge from one ear alone, though there is suppuration in the other also, only the matter being thick and small in quantity does not run out. This shows the necessity in every case of testing both ears as to the distance at which they hear a watch, and of exploring their organic condition.

Of the 2000 cases, one ear only was affected in 361, and both ears in 1639 = 1 : $4\frac{1}{2}$. Of the 361 cases of unilateral disease, the left ear was affected in 167, and the right in 194.

*Auricle.* Of the 5 cases of disease of the auricle, the left ear was affected in 2, the right in 1, and both in 2.

*External auditory passage.* Of the 281 cases of disease of the external auditory passage, the left ear was affected in 54, the right in 62, and both in 165. Of the particular diseases of the external auditory passage, it is to be observed, that phlegmonous inflammation of the auditory passage occurred without exception on one or other side only, that inflammation of the tegumentary lining of the auditory passage and ceruminous glands (catarrhal inflammation), and that periostitis and caries of the auditory passage occurred oftener on one side than on both = 2 : 1, but still often enough on both.

*Membrana tympani.* Acute inflammation of the membrana tympani occurred 21 times in the left ear, 22 times in the right, and twice only in both at the same time.

In the diseases of all the other parts of the ear both sides were much more frequently affected than one only. This was remarkably so in Dr. Kramer's nervous deafness, of the whole number of cases of which 44 only were unilateral, whilst 984 were bilateral = 1 : 22.

If we compare the extraordinary rarity of cases of acute inflammation of the membrana tympani on both sides, viz. 1 : 22 with the great frequency of chronic inflammation of both sides at the same time, viz. 2 : 1, it is evident that the latter inflammation can by no means frequently occur as a sequela of the former, but that the chronic inflammation most commonly comes on unpreceded by an acute stage. Indeed it is probable that acute inflammation has little tendency to pass into the chronic form.

Table iv.—*Of Tinnitus Aurium.*

Of the whole 2000 cases of disease of the ear, there was tinnitus in 1267, and none in 733. All the deaf and dumb were free from tinnitus.

In cases of accumulation of wax in the auditory passage, in acute inflammation of the membrana tympani, and in Kramer's nervous deafness—all essentially different diseases—tinnitus occurred on an average in 3 cases out of 4; whilst in catarrhal inflammation of the auditory passage, in phlegmonous inflammation of the same, in *otitis interna,* and in muculent obstruction of the middle ear, tinnitus was as often absent as present. In chronic inflammation of the membrana tympani, tinnitus was absent twice as often as it was present.

Next to dullness of hearing, tinnitus is the most common complaint of the ear patient. It is very rare that tinnitus occurs without some change in the power of hearing.

Tinnitus varies much in regard to the character of the sound, and in regard to its continuance or intermission, its early or late occurrence in the disease or its period of cessation. But all the varieties of tinnitus may accompany without distinction all the individual diseases of the ear; whilst, on the other hand, almost every disease of the ear may through its whole course remain free from it; and that without any probable reason for its presence in one case or its absence in another. Tinnitus thus appears not to have the slightest value pathognomonically, prognostically, nor even, unless Dr. Kramer's nervous deafness be in some degree excepted, therapeutically.

In accumulations of wax in the auditory passage tinnitus sometimes exists, sometimes not. When present, it subsides along with the deafness on the removal of the wax. The same is the case in muculent obstruction of the middle ear.

Under these circumstances, it is not to be wondered at that similar anomalies as to presence or absence of tinnitus in Kramer's nervous deafness should occur. Dr. Kramer has therefore found reason to give up the division of nervous deafness he formerly adopted, viz. with or without tinnitus, or erethitic and torpid, and to comprehend both under the head of simple nervous deafness.

Table v.—*States of the membrana tympani in chronic inflammation of it.*

Of the 397 cases of chronic inflammation of the membrana tympani, both ears were affected in 279, and one ear only in 118; there were thus 676 individual tympanic membranes in a state of chronic inflammation observed.

In 118 cases out of the 397, i. e. scarcely 1 in 3, the disease was unaccompanied by polypi or perforation, but in the remaining 279 cases, i. e. more than 2 in 3, the membrana tympani was either perforated (217 times), or beset with polypous growths (99 times), or in both states at the same time (37 times). Now as most discharges from the ear (6-7th) depend on chronic inflammation of the membrana tympani with perforation or polypus, the prognosis and treatment of the discharge resolve themselves into the prognosis and treatment of the diseased membrana tympani.

It is oftener the case that the membrana tympani is perforated (129 : 88) or beset with polypi (75 : 24) in one ear only than in both at the same time. When one ear only is thus affected, it is generally the right, much more rarely the left (77 : 52 and 44 : 31). This is in accordance with the fact above pointed out, that the right ear more frequently suffers alone than the left.

Polypi are very rarely observed along with perforation of the membrana tympani 37 : 397 = 1 : 10½.

Perforations of the membrana tympani are very seldom so fine as a pinhole ($\frac{1}{6}$), more frequently they are of the size of a pea or bean ($\frac{2}{6}$), in which case a mere sickle-shaped stripe of the membrane remains. Most commonly the perforations are as large as lentils ($\frac{3}{6}$).

Of 305 cases of perforation, tinnitus was absent in 188—more than the half—and was present in only 117. Proportionally it occurred most frequently when the perforation was of the size of a bean (3 times in 5); somewhat less frequently when the perforation was as small as a pinhole (3 in 7), most rarely when the perforation was of the size of a lentil (3 in 9).

Table vi.—*Simultaneous occurrence of several diseases of the ear.*

Dr. Kramer found one and the same ear affected with more than one disease in 38 cases only out of the whole 2000, which is 1 : 52 ; and the two ears of the same person affected with two different diseases in 66 cases out of the whole 2000 which is 1 : 43.

The diseases most commonly found, the one in the one ear, the other in the other, were :—1. Accumulation of wax in the auditory passage on one side, and Dr. Kramer's nervous deafness on the other (5 times). 2. Chronic inflammation of the membrana tympani on one side, and muculent obstruction of the middle ear (10 times), or nervous deafness (18 times) on the other.

The diseases most commonly found in company in the same ear were as follows: 1. Accumulation of wax in the auditory passage and muculent obstruction of the middle ear occurred only twice in combination in 213 cases of the one, and 164 cases of the other. 2. Accumulation of wax with nervous deafness in one and the same ear occurred 16 times in 1028 cases of nervous deafness, which is 1 : 62. In nervous deafness, it is to be remembered that ordinarily the ceruminous secretion and also the mucous secretion of the middle ear are diminished. 3. Catarrhal inflammation of the auditory passage may be accompanied at the most by a slight degree of inflammation of the membrana tympani, never ending in suppuration, ulceration, or other change of structure.

In 305 cases of chronic inflammation of the membrana tympani with perforation, *otitis interna* occurred in six cases.

In inflammation of the membrana tympani without perforation, the middle ear never was at the same time affected with muculent obstruction.

In erysipelas of the auricle the swelling closes the entrance of the auditory passage, but the inflammation extends no farther.

Accumulation of wax is not accompanied by any disease of the membrana tympani, however long it may have existed, except at the most some slight congestion, which disappears spontaneously. Indeed, reappearance of wax is a good indication of the subsidence of chronic inflammation of the membrana tympani.

In phlegmonous inflammation of the auditory passage, the swelling completely closes the passage, so that no examination of the membrana tympani can be made; but after the abscess has burst, the membrana tympani is found for a short time dim and impaired in transparency.

Inflammation of the mucous membrane of the tympanum with muculent accumulation, Dr. Kramer says, does not spread either to the membrana tympani or to the labyrinth. As soon as the mucus is evacuated the hearing, he says, is restored. He has satisfied himself in more than 164 cases of the kind under notice of the perfect soundness of the membrana tympani. As little is increased secretion succeeded by too great dryness.

Table vii—*Exhibits the situation and mode of life,* and Table viii, *the country and residence* of Dr. Kramer's 2000 patients, but nothing of any consequence admits of being elicited from them.

Table ix.—*Age at which Dr. Kramer's patients became affected.*

From this table it appears that of the 2000 cases of diseases of the ear 504 came on in the first ten years of life = 1 : 4. Of these 504 cases 241 or a half, were cases of chronic inflammation of the membrana tympani; of the whole number of cases of chronic inflammation of the membrana tympani, viz. 397 observed, 2-3ds had their origin in the first ten years of life.

In the second decennial period fewer diseases of the ear have their origin, but in the third decennium the number again increases. From this period the frequency of first attacks of disease of the ear rapidly diminishes, so that from 60 to 70 there is scarcely a single new attack.

The individual diseases made their first attack at the periods of life as follows:

*Accumulation of wax in the auditory passage.* This most frequently first occurred at the age of from 20 to 40; most rarely before the 10th year and after the 60th.

*Catarrhal inflammation of the auditory passage.* Most common below 40, less common after.

*Phlegmonous inflammation of the auditory passage* most common about 20.

*Caries of the auditory passage* in most of the cases occurred in the first decennium, and was dependent on the scrofulous diathesis.

*Acute inflammation of the membrana tympani,* which is quite independent of any diathesis, was most frequent from 20 to 40.

*Chronic inflammation of the membrana tympani,* always dependent on the scrofulous diathesis, occurred in the proportion of 2-3ds in the first decennium, and of this in the first two years—excited by the exanthemata. This thus does not generally arise from an acute attack, but comes on as chronic from the first.

*Muculent obstruction of the cavity of tympanum* comes on especially in the first decennium (2-5ths). In youth it occurs in the proportion of 1-5th, but diminishes after the 20th year. It is always from the first accompanied by great deafness; as both ears are generally at the same time affected, the deafness is very troublesome. By-and-by one ear becomes somewhat free, = 1 : 6, whilst the other remains more obstructed.

*Inflammation of the mucous membrane of Eustachian tube with stricture* most commonly affects one side only (1 : 2), and does not come on so quickly as muculent obstruction; therefore the precise time of origin is not always ascertained.

*Nervous deafness* (1 : 8) also comes on slowly, so that its precise time of origin is not certain; thus differing from catarrh of the middle ear. It would appear first to come on most commonly in the third decennium from violent cold, mental affections, &c. Within the first ten years and after the 60th year, the disease comes on for a first time very seldom (1 : 171). It first begins in one ear, proceeds slowly, and only after a long time perhaps, affects the other.

TABLE X.—*Age of the patients at the time they first consulted Dr. Kramer.*

It is only in the case of diseases attended either by severe pain, or by great and sudden deafness, that the age at the time of consultation corresponds with that at the time of attack. In the other cases a more or less considerable time had been allowed to elapse before the patients applied for assistance.

TABLE XI.—*Duration of the diseases at the time of consultation.*

From this table it is quite evident that by far the largest proportion of diseases of the ear are decidedly chronic in their nature. Only 164 patients out of the whole 2000, i. e. 1-12th, had, at the time of their applying to Dr. Kramer, laboured under the complaint for a period less than four weeks; 1650 had been already labouring under the complaint for a year and more.

Only three diseases of the ear can be considered acute, viz. erysipelas of the auricle, acute inflammation of the membrana tympani, and phlegmonous inflammation of the walls of the auditory passage; all the other diseases have either a decidedly chronic course, or have a tendency to become acute only under particular circumstances, such as catarrhal inflammation of the external auditory passage.

TABLE XII.—*This table shows the different distances at which Dr. Kramer's* 2000 *patients heard the ticking of a watch.*

It is of consequence to have some standard whereby to measure the power of hearing. The ticking of a watch is the most convenient for the purpose. By a sound ear a loud-ticking watch may be heard at the distance of thirty feet. Persons who do not hear the ticking of a watch at all, even when the watch is directly applied to the ear, hear what is said to them only when the speaker is quite close to them and speaks loud, and it is little better even when the watch is heard when directly applied to the ear. It is only when the hearing distance of the watch is as much as inches and feet that the power to follow conversation is at all possessed. Here it is to be remembered that the susceptibility of the ear to the human voice is not always in proportion to its susceptibility to the ticking of a watch. The power to follow conversation is sometimes greater than might be supposed indicated by the shortness of the distance at which a watch is heard; but, on the other hand, it is also sometimes considerably less.

In regard to the diagnostic value of the distance at which a watch is heard, it is to be observed that of itself it is of little importance; more importance is to be attached to the nature of the disease than to the degree of accompanying deafness at the time.

Though the patient may complain of defective hearing on one side only, the surgeon ought not to omit to examine the other, for it will generally be found more or less affected also. See Table III.

In Dr. Kramer's 2000 patients both ears—therefore 4000 ears—were examined as to their hearing distance, but as in 361 cases one ear only was affected, the sound ears of those 361 cases must be deducted from the 4000, when there remain 3639 morbid ears.

From the table, it appears that in all diseases of the auditory passage, membrana tympani, middle ear, and internal ear, a great degree of deafness may occur, but it is in disease of the internal ear that it is most frequent. In the two cases of obliteration of the Eustachian tube, the watch was no longer heard.

*Otitis interna,* inasmuch as it occasions complete disorganization of the organ, was in 3 of the 4 cases most destructive to hearing.

*Dr. Kramer's nervous deafness.* Of the 1028 patients affected with nervous deafness, 214 no longer heard the watch with either ear. Of these 1 only was under 10 years of age, but having learned to speak before the deafness came on, he still retained the faculty.—151 patients no longer heard the watch with one ear, and of these it was the left ear in 77, and the right ear in 74. Those cases in which there is nothing to be hoped for from treatment amounted to 365, which is 1-4th of all the cases of nervous deafness, and of them one half was below 40 years of age.

Of the whole number of affected ears, viz. 3639, or of the whole number of ears affected with nervous deafness, viz. 2012, 217 ears (1 : 9) still heard the watch on immediate application, but in these cases the prospect of improvement is scarcely more favorable than in the former.

339 ears heard the watch at the distance of an inch, and 595 at the distance of a foot. In these degrees of nervous deafness, experience has taught Dr. Kramer that what may be considered as *a very good result of* 3 *to* 6 *months' treatment,* is an increase of the hearing distance from 1 inch to 1 foot in the one set of cases, and from 1 foot to 3 feet in the other.

*In chronic inflammation of the membrana tympani,* though it most generally occasions very considerable organic changes, the highest degree of deafness is only half as frequent as it is in nervous deafness. The lesser degrees of deafness, viz. 1 foot to 3 feet, are the most frequent, one half being about 1 foot and 1-6th about 3 feet. At the same time, however, it is to be observed that the remarkable fact comes out, that with apparently similar organic changes in the membrana tympani there is great difference in the accompanying deafness, and *vice versâ,* with very striking differences in the state of the affected membrana tympani, the same degree of moderate or great deafness may occur. This contradiction is especially frequent when there is perforation of the membrana tympani, and manifests itself here by the impossibility in which we find ourselves to recognize and determine the changes which, in addition to perforation of the membrana tympani, may exist in the parts contained in the cavity of the tympanum.

In chronic inflammation of the membrana tympani in whatever degree, when the patient does not hear the watch, or even when he hears it on application or at a very short distance, there is little or no hope of restoration of hearing even though the inflammation be removed. The prognosis is more favorable only when the hearing distance is several inches, though the prospect of improvement is not so certain in this case, as the impossibility of improvement is in the former.

In periostitis and caries of the auditory passage and in stricture of the Eustachian tube, the highest degrees of deafness are very frequent and are always in exact proportion to the greatness of the organic change of the parts.

But in phlegmonous inflammation of the auditory passage; acute inflammation of the membrana tympani; catarrhal inflammation of the auditory passage; accumulation of wax in the auditory passage; and muculent obstruction of the middle ear, the highest degrees of deafness more rarely occur, notwithstanding very considerable changes of the parts. The highest degrees of deafness, when they do occur, have just as little influence on the prognosis as all the other degrees of impaired hearing. All the diseases under notice are not only decidedly and fully curable, but also the accompanying deafness in whatever degree; the hearing distance is of no consequence in assisting the prognosis, but its progressive increase indicates the progress of the cure.

Table XIII.—*In chronic inflammation of the membrana tympani the watch was heard as follows:*

*a. Chronic inflammation with perforation.* In 217 patients there were 305 membranes in this state; of these 305 affected ears, there was total deafness in 50, an inch of hearing distance in 80, a foot hearing distance in 113, three feet hearing distance in 50, above three feet hearing distance in 9, and undetermined hearing distance in 3.

*b. Chronic inflammation without perforation.* In 180 patients there were 359 ears affected; of these 359 ears there was total deafness in 42, one inch of hearing distance in 88, one foot of hearing distance in 148, three feet of hearing distance in 51, above three feet in 19, and undetermined in 11.

Total deafness occurred proportionally most frequently, viz. in 1-5th, among cases of small sized perforation of the membrana tympani, less frequently, viz. 1-6th, in cases of perforation the size of a lentil, most rarely, viz. 1-8th, when there was no perforation of the membrana tympani.

A hearing distance of an inch was on an average as frequent in the different degrees of perforation, as in non-perforation of the membrana tympani $= 1 : 4$; whilst the hearing distance of a foot was proportionally most frequent among the cases in which the membrane was not perforated $= \frac{1}{3} : \frac{1}{2}$.

A hearing distance of about three feet occurs most frequently in cases of considerable loss of substance of the membrana tympani $= 1 : 4\frac{1}{2}$, less frequently in cases of small perforation or non-perforation $= 1 : 7$, least frequently in lentil sized perforation.

A hearing distance of more than three feet occurred most frequently in cases in which the membrana tympani was not perforated = 1 : 18, and in cases in which it was perforated more frequently in those of the size of a lentil = 1 : 23.

From these observations it results, that in chronic inflammation of the membrana tympani, the hearing is more frequently less deteriorated when the membrane is still unperforated than when it is perforated; and, therefore, that there is little prospect of advantage from the operation of perforating the membrana tympani. If, however, the operation should in any case be had recourse to, as large an opening as possible should be made, as it appears that the hearing was more frequently less deteriorated in cases in which the perforation was large than when it was small. It is still at the same time to be remembered, that in cases of lentil-sized perforation of the membrana tympani, the highest degree of deafness occurred four times more frequently than the lesser degrees, so that the prospect of much benefit even from a large opening is very small.

Lastly, it appears from the table that in none of the cases of perforation was the hearing perfectly unimpared. In 9 cases only did the hearing distance exceed 3 feet (in one 12 feet, in another 10 to 30, but not normal). With such degrees of hearing, however, conversation is so readily carried on by the attentive and careful patient, that an unobservant person might be led to suppose that partial destruction of the membrana tympani is consistent with perfectly good hearing, which is not the case, for when the membrana tympani is perforated, the hearing is always more or less impaired.

Table xiv.—*The patients affected with nervous deafness to such a degree that they no longer heard the watch, were aged as follows :*

Of those who no longer heard the watch with either ear, 214 in number, more than a half (109), and of those who no longer heard the watch with one ear, in number 151, less than a half (71), were in the prime of life, below 40 years of age, and with few exceptions in the enjoyment of good general health. Of the persons above 40, many had laboured under their deafness from early life, and therefore may properly be referred to the preceding category. From these facts it would appear that Dr. Kramer's nervous deafness does not depend on old age or general exhaustion.

Table xv.—*The patients at the time of consultation laboured under other diseases besides that of the ear, as follows :*

More than 4-5ths of the patients were otherwise quite well and had hitherto been so, so that the affection of the ear was quite local and independent. Local treatment alone was employed, and indeed there was no indication for general treatment of any kind.

In the remaining fifth there were complications ; the most frequent of which was general nervous weakness, which, however, almost exclusively occurred in accompaniment with nervous deafness, never in inflammation of the mucous membrane of the middle ear.

Inflammation of the mucous membrane of the middle ear, on its part, very frequently occurs in combination with scrofula; (there were 26 cases of this complication in the whole 164 cases of muculent obstruction of the middle ear, or 1-6th;) and with catarrh (there were 18 cases of this complication or 1-9th). Catarrh did occur along with nervous deafness but rarely.

In the cases of inflammation of the glands of the auditory passage, the scrofulous diathesis occurred in the proportion of 1 : 8. In chronic inflammation of the membrana tympani it occurred in the same proportion. Gout and rheumatism were rare.

Catarrh was most frequent in cases of muculent obstruction of the middle ear, and especially in stricture of the Eustachian tube. It did occur but rarely in nervous deafness.

Vertigo occurred in 1 case of accumulation of wax, in 3 cases of polypus of the membrana tympani, and in 7 cases of nervous deafness.

Hemorrhoids occurred in 7 cases of nervous deafness.

Phthisis in 1 case of caries of the middle ear, and in 4 cases of nervous deafness.

In the acute inflammations there was more or less inflammatory fever.

TABLE XVI.—*Causes of the diseases of the ear in Dr. Kramer's* 2000 *patients.*

The actual commencement of many of the diseases of the ear is frequently quite unobserved, pain seldom occurring; and as regards discharge, deafness, and tinnitus, they often occur only after the proper disease has already existed some time. Attention not being paid to this circumstance, and the discharge, deafness, or tinnitus being taken as a manifestation of the commencement of the disease, influences to which the patient may have been accidentally exposed at the time of the occurrence of those symptoms, are often put down as the cause of the disease. Hence the determination of the causes of diseases of the ear is often very difficult.

In more than one half of the whole 2000 cases, viz. in 1109 the cause remained undetected.

Hereditary predisposition to nervous deafness is not unfrequent, in the proportion of 1 : 6 ; but the same predisposition to muculent obstruction of the middle ear, and to chronic inflammation of the membrana tympani is comparatively rare : the former five times more rare, the latter nine times.

Cold appears to have been the most common cause of acute inflammation of the membrana tympani (2-3ds), and phlegmonous inflammation of the auditory passage (one half). It is also a frequent enough cause of the inflammation leading to muculent obstruction of the middle ear (1-3d), of the erythematous inflammation leading to accumulation of wax in the auditory passage (1-3d), and of the inflammation of the glands of the passage (1-3d). It much more rarely happens (1-10th) that cold can be with certainty determined to have been the cause of Dr. Kramer's nervous deafness, and of chronic inflammation of the membrana tympani (1-14th).

The exanthemata, and other diseases of the skin, are frequent causes of chronic inflammation of the membrana tympani, especially scarlet fever, this having been the cause of 1-4th of all Dr. Kramer's cases of chronic inflammation of the membrana tympani.

Only 1-68th of all Dr. Kramer's cases of nervous deafness appear to have had their origin in scarlet fever.

Nervous and gastric fevers sometimes though rarely call forth chronic inflammation of the membrana tympani and nervous deafness. Two cases of muculent obstruction of the middle ear appear to have been called forth by gastric fever; when thus excited these diseases were much developed and proved very obstinate.

Blows on the ear excited inflammation of the glands of the auditory passage in three cases. Chronic inflammation of the membrana tympani in 12, and nervous deafness in 24. Nervous deafness was also in some cases instantaneously produced in the greatest degree by injury of the head and spine.

Disease of the brain with convulsions in infants, was a common cause of deaf dumbness (1-3d).

Great noise, sorrow, severe toothache, exhausting hemorrhage, were sometimes the cause of nervous deafness.

TABLE XVII.—*Perforation of the membrana tympani occurred after scarlet fever, measles, cold, and smallpox,* thus:

Chronic inflammation of the membrana tympani, excited by cold or smallpox, most frequently in proportion led to perforation, viz. in the former case 22 : 29 = 3 : 4, in the latter 9 : 11 = 3 : 4, more rarely when it occurred after scarlet fever 49 : 89 = 1 : 2, or measles 15 : 28 = 1 : 2.

It is the converse of what has now been stated, when the question is, by what cause is perforation on both sides most frequently occasioned. Perforation on both sides occurred most frequently after measles, 14 times in 15 cases, somewhat more rarely after scarlet fever 32 : 49 = 2 : 3, still more rarely after smallpox 3 : 9 = 1 : 3, most rarely after cold 6 : 22 = 1 : 3½.

As to the extent of injury of the membrana tympani from the inflammation excited by the different causes under notice, it was proportionally greatest after smallpox: of 12 cases the perforation was in 5 the size of peas; more rarely of this size after measles, 8 times in 29 cases; after scarlet fever, 20 times in 81 cases; and from simple cold, 7 times in 28 cases.

Smallpox is thus most destructive of the membrana tympani, as it is the most frequent cause of perforation, and that of the greatest extent. Measles again is the most frequent cause of perforation of the membrane on both sides at the same time. Scarlet fever and cold were somewhat less destructive, but still destructive enough.

TABLE XIX.—*Results of Dr. Kramer's treatment of his* 2000 *patients.*

From this table it is seen that the curability and improvability of the different diseases of the ear is very different.

That cure or improvement of the organic lesion has taken place admits of being determined, in the case of the auricle, by direct inspection,—in the case of the auditory passage and membrana tympani, with the assistance of the *speculum auris,*—and in the case of the cavity of the tympanum and Eustachian tube, by means of catheterism and the air-douche. That cure or improvement of the hearing has taken place, is of course determined by the hearing distance. In the nervous deafness of Dr. Kramer, this is the only point which admits of being determined.

If in order for the cure or improvement of a disease of the ear to be admitted, its permanency be required, this is, says Dr. Kramer, altogether *unreasonable;* for no treatment in the world can render any diseased organ of the body, and therefore the ear, unsusceptible to renewed attacks.

Dr. Kramer evidently makes these remarks to meet the objection that cure or improvement of disease of the ear is very often not permanent. However true the remarks may be, the objection is not practically removed; what a patient desires, is to have his disease permanently cured or relieved, and in the hope of this he subjects himself to the trouble and expense of treatment. But what is the advantage gained, if after the cessation of the treatment he quickly relapses into his former state?

As regards curability, Dr. Kramer divides diseases of the ear into four groups, viz. 1, The unconditionally curable; 2, The conditionally curable; 3, Those conditionally capable of amelioration only; 4, The unconditionably incurable.

1. *The unconditionally curable, notwithstanding long continuance, neglect, and very considerable development of the disease.* Under this head, Dr. Kramer places erysipelas of the auricle, boils of the auricle, accumulation of wax in the auditory passage, catarrh of the auditory passage, phlegmonous inflammation of the auditory passage, acute inflammation of the membrana tympani, catarrh with muculent obstruction of the middle ear.

*Erysipelas and boils* of the auricle do not require any particular remark.

*Accumulation of wax and exfoliated epidermis in the auditory passage.* The erythema of the integument of the auditory passage in which this originates, quickly passes off and does not become the subject of treatment. The plug of wax and exfoliated epidermis are readily removed.*

On the removal of the accumulation of wax and epidermis, there is in general immediate and complete cure of the deafness, tinnitus, confusion of the head, &c., with which the patient had been troubled. Any redness of the skin of the auditory passage and membrana tympani which may exist, spontaneously disappear. In Dr. Kramer's table there are 213 cases of such immediate and complete cure after, in some of them, whole years' continuance of the complaint.

Sometimes, though not often (16 times in the 2000 cases), along with the accumulation of wax in the auditory passage there is nervous deafness. In such a case of course the removal of the accumulation effects relief of the deafness, tinnitus, &c., only so far as these depend on the accumulation. After the removal of the wax the case in respect of its curability comes under Dr. Kramer's second group.

*Catarrh or inflammation of the skin and glands of the auditory passage,* always yields to acetate-of-lead lotions, counter-irritation, and gentle purgatives, and in recent cases very quickly. The disease is not so tractable when there is a disposition to skin and glandular disease generally. Besides the local, the appropriate general treatment is necessary, and months may be required for the cure. Of 51 cases of this kind 49 patients were eventually cured, whilst in the 2 others the patients withdrew themselves from treatment before a cure was effected.

*Phlegmonous inflammation of the auditory passage.* If this is not resolved by the application of leeches, it comes in the course of a few days under the application of emollient cataplasms to suppuration; and when the abscess bursts the symptoms quickly subside. In consequence of the closure of the auditory passage by the swelling, hearing is disturbed for a time, but it is completely regained on the subsidence of the swelling.

* To this, which is all that Dr. Kramer says, we would add, that sometimes, in addition to the exfoliation from the walls of the auditory passage, there is exfoliation of epidermis from the membrana tympani in numerous layers, which, being tinged brown with unhealthy wax, looks like a plug of wax deep in the passage. It sometimes adheres closely to the membrana tympani, and does not admit of being readily removed, and no attempt should be made to remove it. By and by, it becomes loose and comes away.

*Acute inflammation of the membrana tympani.* This generally yielded in a few days to leeches and counter-irritation, and acetate-of-lead lotions. Repeated leeching was necessary in severe cases; 1 case only out of 45 was longer than eight days under cure.

*Inflammation of the mucous membrane of the middle ear with muculent obstruction.* All his cases of this complaint, Dr. Kramer tells us, were without exception perfectly cured "by dispersion of the mucus, and by bringing the secretion of it back to the natural standard." To this result the local treatment by catheterism and the air-douche contributed most. In recent cases blowing through the catheter merely, was sufficient without using the air-press.

The subjects of most of these cases, we would observe, were young, and there can be little doubt that the recent cases at least would have recovered without catheterism, merely by the patient making expiratory efforts with the nose and mouth closed.

When the muculent obstruction has lasted long, or when the accumulated mucus is so viscid that it admits of being only partially dispersed, Dr. Kramer found that the deafness was only partially relieved, and that the mucus collected again very soon with consequent aggravation of the deafness. In such cases Dr. Kramer found it necessary to use the air-press to disperse the mucus. At the same time he observes that the *cold air* gave tone to the morbid membrane!

In patients with the scrofulous diathesis, Dr. Kramer, besides using the local treatment just mentioned, ordered appropriate diet and medicines.

2. *Conditionally curable diseases.* The conditions on which the curability in these cases depends, are slight development, short continuance, &c., of the disease. Under this head, Dr. Kramer places eczema of the auricle, periostitis, and caries of the osseous auditory passage, chronic inflammation of the membrana tympani, stricture of the Eustachian tube, nervous deafness.

Passing over *eczema of the auricle* and *caries of the osseous part of the auditory passage,* both of which are stubborn complaints, and not in their nature and treatment peculiar to the ear, we come to:

*Chronic inflammation of the membrana tympani.* When this, as is often the case, is connected with a dyscrasic state of constitution, its curability depends much on the curability or improvability of the latter. Its curability, however, depends in a much greater degree on the extent of organic change which the membrane has undergone. When the membrane has once become much thickened or perforated, there is no hope of a restoration of its natural structure. Polypi, which for the most part have their origin in chronic inflammation of the membrana tympani, may indeed generally be removed, except when they are broad, flat, and sessile; but, as the membrana tympani, at the same time that it is the part where the polypus has its root, is the seat of the other changes above mentioned, viz. thickening or perforation; the removal of the polypus does not effect very material improvement.

If, therefore, says Dr. Kramer, it be taken into consideration, that in one half of the whole number of cases of chronic inflammation of the membrana tympani, there was perforation of the membrane, and that in one fourth of the whole number of cases there were polypous growths, it is not to be wondered at that I have succeeded in perfectly curing 1-14th only of the whole number of cases; and in improving only more or less 11-14ths so far that the suppuration was moderated, seldom quite removed, and not unfrequently the hearing somewhat improved. Two fourteenths of all the cases remained uncured.

*Stricture of the Eustachian tube.* The figure of the curability of this affection is still more unfavorable than that of the curability of chronic inflammation of the membrana tympani.

*Nervous deafness.* "As the little success of treatment," says our author, "in chronic inflammation of the membrana tympani and in stricture of the Eustachian tube is essentially owing to the invariable obstinacy of the organic changes of the affected parts, so in nervous deafness the results of treatment have been equally unfavorable in consequence of the too far advanced state of depression of the vital energy of the auditory nerves. They, in fact, not only are affected with simple diminished susceptibility for sound, even to complete deafness, but at the same time with a morbid susceptibility for impressions of every kind, increasing with the deafness. The consequence of this is, that the mildest applications only can be borne. Indeed in cases of much developed nervous deafness, the impossibility of any improvement depends principally on the impossibility of finding remedies which will operate gently enough on the auditory nerves and not overirritate them." (pp. 179-80.)

It will be seen that these therapeutical speculations of our author are dictated by the pathological views he entertains regarding the cases he calls cases of nervous deafness, but from which views we have above altogether dissented. Practically, however, we equally admit the hitherto little curability of the affections referred to.

Besides these extremely unfavorable circumstances, Dr. Kramer encountered still another, viz., that 3-5ths of his patients labouring under nervous deafness had already passed their 30th year before consulting him, and that in more than half, the complaint had already existed longer than 10 years; and lastly, that in 2-7ths the deafness had already attained the highest degree—viz., either total deafness or incapacity to hear the watch on application.

Dr. Kramer either did not at all undertake the treatment, or, having undertaken it, soon relinquished it as hopeless in 5-20th, i. e. 271 of his patients, in consequence of their not bearing the mildest applications, although the circumstances of the case were otherwise favorable, such as youth and a moderate degree of deafness.

On the other hand, he succeeded in benefiting, both in regard to hearing and tinnitus, 14-20ths of his patients, i. e. 703, though in very different degrees, according to the age of the patient, the length of time the disease had existed, the degree of its development, and to the length of the treatment.

In 1-20th, or 54 of the cases only, was a perfect cure effected, but the attending circumstances were of the most favorable kind, such as inconsiderable development of the disease and youth of the patient.

In regard to his mode of treating nervous deafness, Dr. Kramer tells us that it is still essentially the same as that described in his treatise on the Nature and Treatment of the Diseases of the Ear, viz. the introduction into the cavity of the tympanum of stimulating vapours. Instead of the vapour of acetic ether, however, which he formerly recommended, but which he has of late years found too stimulating, and therefore not so well borne, he recommends the vapour of *aqua assafœtidæ simplex,* of musk, of *aqua amygd. amar. sine spiritu parata,* and the like.

General treatment, in addition to this local treatment, he had recourse to only for other attendant complaints, the occurrence of which was on the whole rare. But he remarks, that although such concomitant complaints be removed by the general treatment, the nervous deafness may be little or not at all benefited. It is, however, he continues, always quite proper, before the commencement of the local treatment of the auditory nerve, to bring the general health into as good a state as possible.

3. *Diseases incapable of cure, but under peculiarly favorable circumstances admitting of amelioration.* Under this head Dr. Kramer places *otitis interna.* In otitis interna resolution sometimes takes place, but seldom. In most cases, besides destruction of the organ, there is danger of life.

4. *Unconditionally incurable diseases.* Under this head Dr. Kramer places obliteration of the Eustachian tube and deaf-dumbness.

Besides the "Statistics," of which we have now given a very full abstract, the work before us contains three other contributions, viz. on the Acoustics of the human ear, on Electro-magnetism as a remedy for dullness of hearing and tinnitus aurium, and on Otorrhœa cerebralis. The first and the last of these we pass over, but pause to give the following results of the experience of Dr. Kramer and others as to the value of electro-magnetism as a remedy in deafness and tinnitus aurium.

1. The magneto-electric or electro-magnetic current acts decidedly as an excitant on the organ of hearing.

2. This action is strongest when there is an affection of one or both auditory nerves, and the magneto-electric current is conveyed from the mouth of the Eustachian tube to the external auditory passage of the same ear, instead of from one auditory passage to the other.

3. The exciting action of megneto-electricity is manifested by convulsive twitchings, pains in the ear, momentary increase of the hearing distance (which, however, very soon diminishes again, or, though it continues some time, it does not amount to any great degree), and by aggravation of the tinnitus, either at the moment or some time after the sitting.

4. Magneto-electricity or electro-magnetism does not thus appear to possess any peculiar strengthening action on the auditory nerves. On the contrary, great care is necessary in the employment of the remedy not to over-excite the affected auditory nerves.

5. Hitherto the results of experience as to the use of the rotation apparatus in diseases of the ear are not encouraging. If, however, for any reason it be had recourse to, nervous deafness is the complaint for which it is most adapted; but that the complaint is nervous deafness ought to be first determined by careful instrumental exploration of the outer and middle ear.

6. The application of magneto-electricity ought always to be made with the greatest precaution and in the mildest manner, and always given up whenever, notwithstanding every precaution, decided aggravation of tinnitus takes place, and improvement of the hearing distance is not effected, or, when effected, it is again lost.

